# Presumptive Neurocysticercosis with Concurrent Bacterial Infection: A Diagnostic Challenge

**DOI:** 10.3390/antibiotics14121205

**Published:** 2025-12-01

**Authors:** Martina Di Giuseppe, Lucia Scarlato, Lorenza Romani, Laura Cursi, Chiara Carducci, Maia De Luca, Sara Chiurchiù, Davide Luglietto, Giulia Lorenzetti, Costanza Tripiciano, Stefania Mercadante, Stefania Bernardi, Carlo Efisio Marras, Laura Lancella

**Affiliations:** 1 Infectious Diseases Unit, Bambino Gesù Children’s Hospital, IRCCS, 00165 Rome, Italy; 2 Pediatric Infectious Diseases Unit, Giovanni XXIII Children Hospital, University Hospital, Bari, Italy; 3 Neuroradiology Unit, Imaging Department, Bambino Gesù Children’s Hospital, IRCCS, 00165 Rome, Italy; 4 Neurosurgery Unit, Bambino Gesù Children’s Hospital, IRCCS, 00165 Rome, Italy

**Keywords:** neurocysticercosis, brain abscess

## Abstract

**Background**: Neurocysticercosis is a parasitic infection of the central nervous system caused by the larval stage of *Taenia solium*. This disease is endemic in some countries in Central and South America, South and South-East Asia, and sub-Saharan Africa. In North America, Europe, Japan, and Australia, only sporadic cases are documented. Moreover, reports of bacterial superinfection arising within neurocysticercotic lesions remain exceptionally scarce. **Methods**: We report a clinically severe and diagnostically challenging case of suspected neurocysticercosis with cerebral streptococcal superinfection in a 17-year-old Italian patient with Down syndrome and no history of travel to endemic regions. **Results**: The patient, with pre-existing epileptic encephalopathy, presented with progressive drowsiness and altered mental status, rapidly deteriorating to cardiorespiratory arrest. Neuroimaging demonstrated multiple ring-enhancing lesions, in conjunction with positive *Taenia solium* serology. *Streptococcus* spp. was identified in one neurosurgically drained lesion, consistent with secondary bacterial involvement in association with concurrent pneumonia. Combined antiparasitic therapy and targeted antimicrobial treatment resulted in sustained clinical and radiological improvement. **Conclusions**: In non-endemic settings, neurocysticercosis should remain within the differential diagnosis of unexplained epilepsy and multifocal CNS lesions. Although rare, bacterial superinfection warrants consideration in atypical presentations, particularly in individuals with concomitant infectious foci and underlying immune dysfunction such as that associated with Down syndrome.

## 1. Introduction

Neurocysticercosis is a parasitic disease resulting from infection of the central nervous system by the larval stage of the pork tapeworm *Taenia solium* [1]. Although *Taenia solium* is a two-host zoonotic cestode, only humans serve as the definitive host for the adult tapeworm, whereas both pigs and humans can act as intermediate hosts harboring the larval form [2]. Humans acquire cysticercosis through the ingestion of *T. solium* eggs from tapeworm carriers via fecal–oral transmission [2]. Therefore, vegetarians and people who do not consume pork can also contract cysticercosis if they are in close contact with infected individuals [3]. After ingestion, eggs release oncospheres in the duodenum. These penetrate the intestinal mucosa and migrate through the lymphatic and vascular systems to various tissues in the body, where they develop into larval form cysticercus over a period of about three months [4]. The most common sites of cysticercus development are the subcutaneous and intramuscular tissues, followed by the brain and eyes [3].

Neurocysticercosis refers to the involvement of the central nervous system by cysticercosis, affecting structures such as the brain parenchyma, ventricular system, basilar cisterns, sulci, gyri, spinal cord, and retina [5]. It represents the most common parasitic disease of the human central nervous system [6] and a major cause of epilepsy in tropical countries [7]. Neurocysticercosis is endemic in many countries, with high prevalence in Central and South America, South and South-East Asia, and sub-Saharan Africa [8], although it is almost non-existent in Muslim countries [9,10]. Conversely in North America, Japan, Australia, and Central Europe, including Italy, only sporadic cases have been reported, mainly in immigrants or travelers from endemic regions [11,12,13]. However, neurocysticercosis remains a public health concern even in Europe, where some autochthonous cases have been reported, highlighting the potential for local transmission [14].

Cysticerci may remain in the central nervous system asymptomatically for prolonged periods. In symptomatic neurocysticercosis, the host immune response is predominantly mediated by the Th1 pathway, leading to granuloma formation [7]. Bacterial superinfection of these lesions is extremely rare but may lead to severe and potentially life-threatening complications. Secondary bacterial infection usually occurs in the presence of immunosuppression, but it has also been reported in immunocompetent patients. Pyogenic organisms typically reach cysts in the central nervous system via the bloodstream, although a systemic source of infection is not always identified [15].

In this report, we describe a severe and clinically complex case involving suspected neurocysticercosis complicated by cerebral streptococcal superinfection in a 17-year-old Italian boy living in Southern Italy, with no history of travel to endemic regions. The diagnostic process was particularly challenging, not only due to the absence of conventional epidemiological risk factors, but also because of the patient’s pre-existing epileptic encephalopathy in the setting of Down syndrome—an underlying condition associated with both atypical neurological presentations and immune dysregulation. Given the lack of histopathological confirmation and the limitations of serological testing, the diagnosis must be considered presumptive. Nevertheless, the constellation of clinical, radiological, and microbiological findings raised a strong suspicion of neurocysticercosis in the context of secondary bacterial involvement. To date, only a handful of cases describing bacterial superinfection of neurocysticercotic lesions have been reported in the literature. Although rare, this complication can lead to rapid deterioration and should be actively considered when multifocal lesions are accompanied by disproportionate perilesional edema and mass effect [15].

## 2. Case Report

A 17-year-old boy was brought to the emergency department for drowsiness and altered mental status. Upon arrival, he developed cardio-respiratory arrest requiring immediate cardiopulmonary resuscitation and orotracheal intubation.

His past medical history was notable for Down syndrome, severe intellectual disability, and epileptic encephalopathy characterized by tonic seizures occurring in clusters. He was on chronic anti-epileptic polytherapy with sodium valproate, levetiracetam, clobazam and cannabidiol. A brain MRI performed at age 3 showed no abnormalities, and no additional neuroimaging studies were obtained prior to this episode.

One month before the current admission, he had been hospitalized for pneumonia treated with intravenous ceftriaxone, without any microbiological isolation or pathogen identification. He lived in Puglia, with no history of travel outside Italy, no contact with livestock, and no reported consumption of raw or undercooked meat or unpasteurized dairy products. The patient was known to frequently place non-food objects in his mouth, a behavior consistent with pica and considered a potential risk factor for inadvertent fecal–oral exposure. Household members were asymptomatic and were not screened with stool testing for *Taenia solium* carriage.

**Figure 1:**
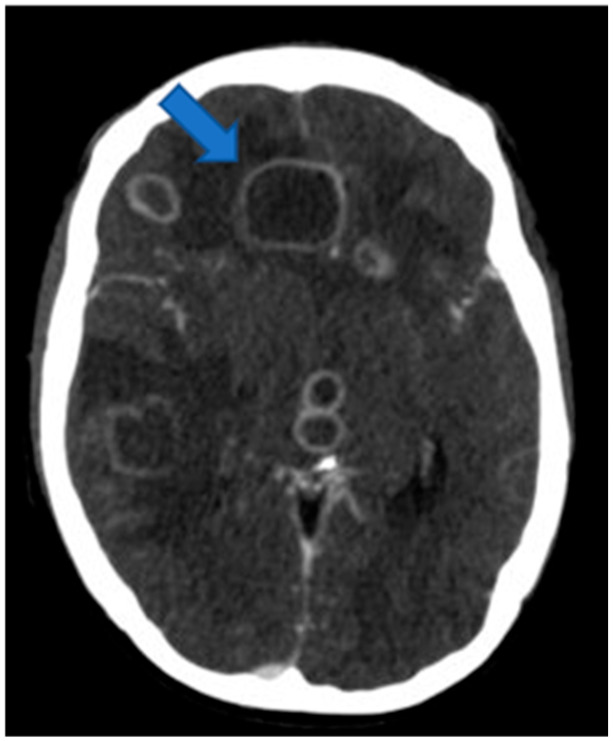
Baseline Axial CT scan: multiple hypodense nodular lesions with marked peripheral contrast enhancement, varying in size; the largest one located in the right paramedian frontal region (Figure 1, arrow).

Diagnostic neuroimaging was performed using a contrast-enhanced cranial CT scan which revealed multiple nodular hypodense lesions with marked peripheral post-contrast “ring” enhancement, ranging in size from a few millimeters to several centimeters. The largest lesions, associated with modest regional mass effect, were located in the right paramedian frontal lobe, right temporal lobe, right thalamic–mesencephalic region, and left temporopolar region (Figure 1). A standard posteroanterior and lateral chest radiograph was obtained using digital radiography and demonstrated left para-retrocardiac pneumonia with a small pleural effusion.

Admission blood tests included complete blood count, C-reactive protein (CRP), renal and hepatic panels. Laboratory evaluation demonstrated neutrophilic leukocytosis and elevated CRP (4 mg/dL; normal < 0.5 mg/dL). Lumbar puncture was performed and cerebrospinal fluid (CSF) was analyzed for cell count, protein, glucose, Gram-Stain, and bacterial cultures. CSF analysis was unremarkable and both CSF and blood cultures were negative.

Upon admission to the intensive care unit, empiric broad-spectrum anti-infective therapy was initiated, consisting of ceftriaxone (100 mg/kg/day), vancomycin (40 mg/kg/day), metronidazole (30 mg/kg/day), and liposomal amphotericin B (3 mg/kg/day).

**Figure 2:**
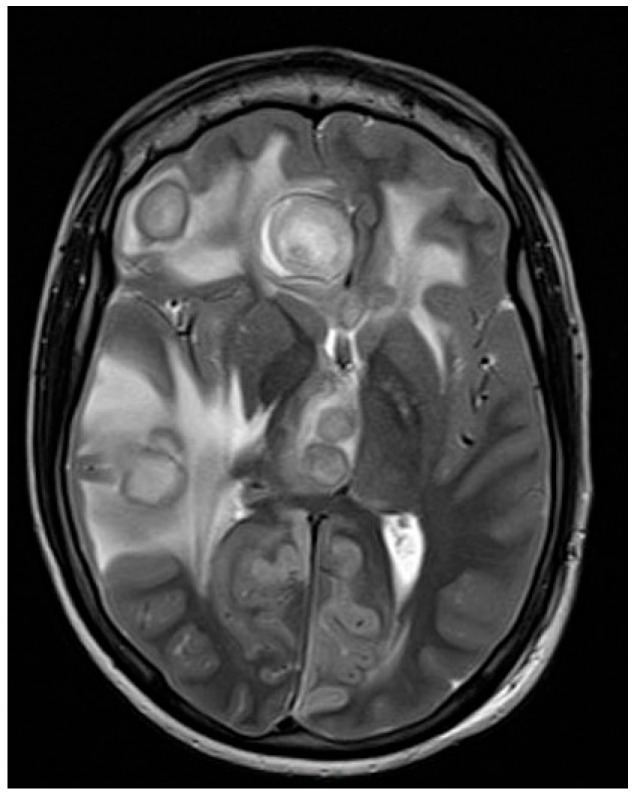
Baseline MRI, multiple focal lesions with a widespread bihemispheric distribution (right > left) and involvement of the right thalamus, accompanied by extensive vasogenic perilesional edema.

MRI was performed within 24 h using a 3 Tesla system with T1-weighted, T2-weighted, FLAIR, diffusion-weighted imaging (DWI), and contrast-enhanced sequences. Imaging confirmed multiple nodular ring-enhancing lesions in both hemispheres, including the thalamic region, with surrounding edema and mass effect (Figure 2).

Given signs of elevated intracranial pressure, neurosurgical intervention was undertaken under general anesthesia, including placement of an external ventricular drain and stereotactic-guided drainage of one lesion. The specimen was submitted for microbiological and histopathological evaluation. The drained material was analyzed with the BioFire^®^ FilmArray^®^ Blood Culture Identification (BCID) panel, a multiplex PCR assay designed to rapidly identify common bacterial and fungal pathogens and selected resistance markers directly from positive cultures. The assay detected *Streptococcus* spp.; subsequent conventional cultures remained sterile, likely due to prior antimicrobial therapy.

Histopathological examination of the drained material included hematoxylin–eosin staining, periodic acid–Schiff (PAS), and Grocott methenamine silver staining. Findings revealed friable necrotic material with dense neutrophilic infiltration; PAS and Grocott stains were negative for fungal elements.

In light of these results, liposomal amphotericin B was discontinued, and antimicrobial therapy was continued.

Serological testing for Taenia solium was performed at admission using a qualitative IgG ELISA (NovaLisa^®^, NovaTec Immundiagnostica GmbH, Dietzenbach, Germany). The test returned positive. Due to the limited amount of surgical material, direct parasitological examination was not feasible. Stool microscopy for *T. solium* eggs was performed using triple-sample concentration technique and was negative. Anti-Toxoplasma IgG/IgM serology was also performed and resulted negative.

Given the coexistence of compatible neuroimaging, positive serology, and bacterial superinfection, we hypothesized secondary infection of cysticerci by *Streptococcus* spp., with pneumonia as a potential source of bacteremia. Antiparasitic therapy was initiated according to established recommendations for multiple viable lesions: albendazole (15 mg/kg/day) plus praziquantel (50 mg/kg/day) for 4 weeks. Dexamethasone (0.1 mg/kg/day) was administered 24 h before antiparasitic therapy and tapered progressively thereafter. Targeted antibacterial therapy was continued for 8 weeks, with adjustments guided by clinical and laboratory response.

**Figure 3:**
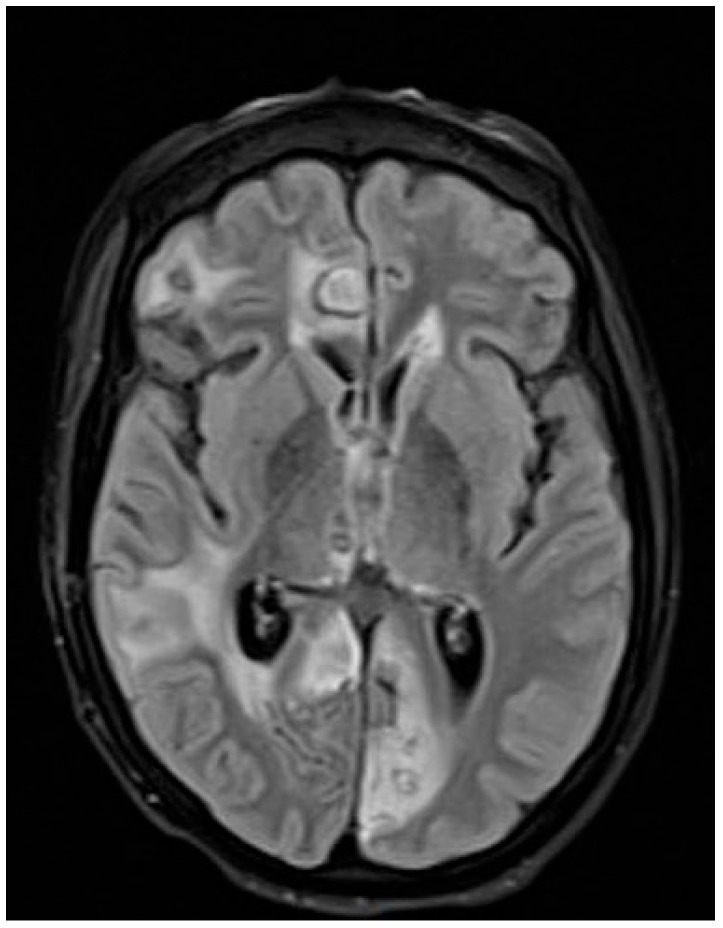
Brain MRI two months after therapy.

**Figure 4:**
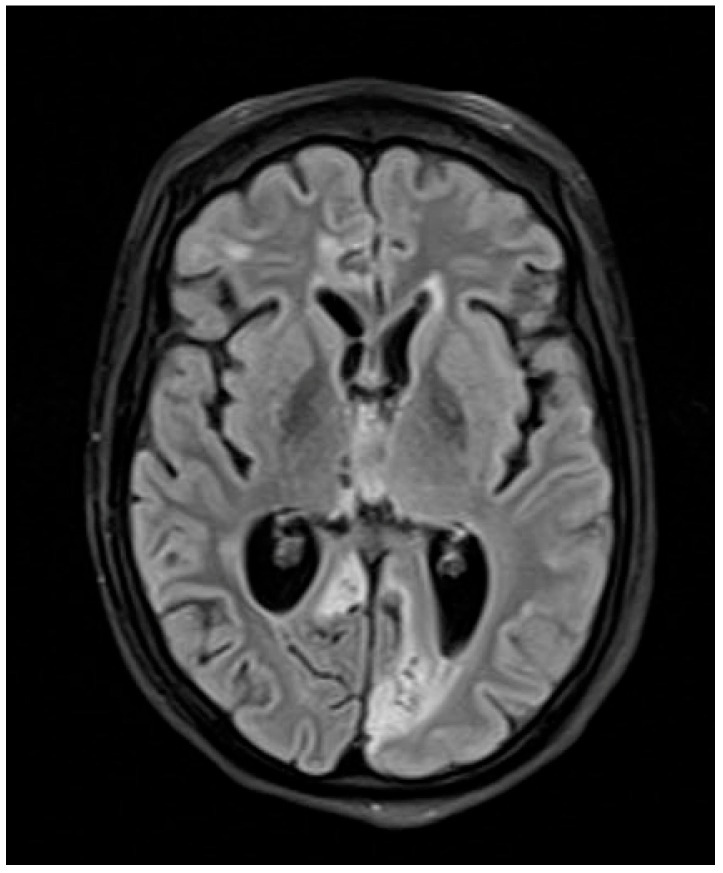
Brain MRI four months after therapy.

The patient showed progressive neurological improvement and was discharged after 10 weeks of hospitalization. Follow-up *T. solium* serology was borderline at 1 month and negative at 2 months. Follow-up MRI at 2 months and 4 months was performed using identical imaging parameters to ensure consistency and demonstrated progressive reduction in lesion size and edema (Figure 3 and Figure 4).

Clinical follow-up was maintained for 4 months, during which the patient’s neurological status remained stable with no recurrence. After this period, the patient was lost to follow-up due to family relocation. A detailed chronological overview of the clinical course is provided in Table 1**.**

## 3. Discussion

Cysticercosis is acquired by ingesting eggs of *T. solium* typically through contaminated food, water, or surfaces, or through direct fecal-oral contact with an infected person hosting the adult tapeworm. In the intestine eggs release oncospheres that migrate through the blood and lymphatic system to the central nervous system, especially in areas with high blood flow such as the gray-white matter junction, where they develop into cysticerci [12]. Here it can manifest with various neurological disorders and for this feature in endemic areas neurocysticercosis is known as “the great imitator” [16]. However, the major presentation is epilepsy: seizures are usually focal or secondarily generalized-focal. Other manifestations of parenchymal brain cysticercosis include focal neurological deficits, chronic headaches, increased intracranial pressure, cognitive decline and psychiatric disorders. Extra-parenchymal neurocysticercosis may present with different symptoms according to the involved areas: obstructive hydrocephalus in ventricular and subarachnoid neurocysticercosis; radicular pain, paresthesia, paraplegia and sphincter disturbances in spinal cysticercosis; visual deficits, acute unilateral blindness or ophthalmoparesis in ophthalmic cysticercosis [7]. Extra-parenchymal neurocysticercosis has a worse prognosis [16].

Despite development in neuroimaging methods and immune diagnostic tests, neurocysticercosis diagnosis remains challenging. Neuroimaging findings have poor specificity, except for visualization of a scolex that is considered pathognomonic. However, according to Del Brutto Et Al. revised criteria, validated for ventricular lesions, the evaluation with both computed tomography (CT) plus MRI increases the likelihood to establish a definitive diagnosis even in the absence of a discernible scolex [1,17].

Laboratory tests may support the diagnosis through the detection of antibodies or antigens related to *T. solium* infection [1,7]. The most reliable test for antibody detection is the enzyme-linked immunoelectrotransfer blot, but it is not always available. On the other hand Enzyme-linked immunosorbent assays (ELISA) are widely available but not recommended for their poor specificity and sensitivity [1]. Nevertheless, quantitative serial serum ELISAs could be useful to monitor the response to antiparasitic treatment in people with extensive disease [16]: in our case ELISAs were positive at the beginning (before treatment), then low detectable until to became undetectable.

In this case, the diagnosis must therefore be regarded as presumptive, as no parasitological or histopathological demonstration of cysticerci was obtained from CNS tissue, and ELISA positivity alone cannot establish certainty.

A comprehensive differential diagnostic evaluation was undertaken. Toxoplasmosis was excluded by negative serology. Tuberculosis, an important cause of multifocal CNS lesions, was considered but deemed unlikely in the absence of epidemiological exposure, compatible pulmonary findings, or microbiological evidence. Fungal infections—cryptococcosis, aspergillosis, mucormycosis—were ruled out on the basis of negative stains and sterile cultures. Primary bacterial abscesses remained plausible given the concomitant pneumonia and detection of *Streptococcus* spp. in the drained lesion; however, the extensive number, bilateral distribution, and size heterogeneity of lesions did not support a purely pyogenic etiology. Neoplastic conditions, both primary and metastatic, were also considered but were excluded based on histological examination, which showed no evidence of neoplastic tissue.

The Del Brutto criteria [18] were intentionally not applied to classify the case as “definitive” or “probable”, because major criteria, including histopathological identification of the parasite and visualization of a scolex, were not fulfilled. Moreover, these criteria were developed and validated in clinical scenarios that did not account for secondary bacterial superinfection, limiting their applicability here.

Epidemiological interpretation also warrants caution. Although the patient had no history of travel to endemic areas, household members were not screened, and asymptomatic tapeworm carriage among close contacts cannot be excluded. Additionally, cysticercosis can remain asymptomatic for years or decades, making early-life acquisition another plausible explanation.

Beyond epidemiology, Down syndrome (DS) provides an important biological and behavioral context for understanding this presentation. DS is associated with impaired neutrophil chemotaxis, reduced T-cell proliferation, and features of premature immune senescence, which collectively compromise both innate and adaptive immunity [18]. These defects diminish host defense against pathogens and weaken containment of infection once microorganisms breach protected compartments such as the central nervous system. Gastrointestinal and behavioral factors, including pica, further increase exposure risk [19] documented a meaningful prevalence of gastrointestinal parasitic infections in individuals with DS, supporting the relevance of environmental and behavioral contributions. Longstanding pica behaviors in this patient provide a credible route for inadvertent fecal–oral exposure.

These immunological and behavioral vulnerabilities also offer a mechanistic rationale for bacterial superinfection of parasitic cysts. Parasitic lesions create locally inflamed tissue, and impaired neutrophil recruitment, reduced adaptive responses, and immune dysfunction may weaken local defenses. DS is additionally associated with increased susceptibility to respiratory infections and aspiration events, providing a plausible source of transient bacteremia capable of hematogenous seeding of inflamed CNS tissue. Concomitant pneumonia in this patient thus offers a biologically credible pathway for Streptococcus superinfection of pre-existing lesions.

An additional layer of diagnostic uncertainty arose from the rapid serological conversion from positive to negative ELISA. This may reflect a false-positive initial result, cross-reactivity with bacterial antigens during acute systemic infection, or laboratory error, and alternative diagnoses cannot be entirely excluded.

Neurocysticercosis treatment is based on antiparasitic drugs, corticosteroids and antiseizure medication. For patients with few lesions (1–2 viable cysticerci), albendazole (15 mg/kg/day) for 8–14 days represents the first line therapy. For patients with more than two viable cystic lesions, as our patient, experts recommend adding praziquantel (50 mg/kg/day) [1]. A longer treatment of 28 days is preferred in children with multiple lesions [7]. Combination with corticosteroids is suggested for patients with numerous cystic lesions associated with inflammatory reaction. However, in case of diffuse cerebral edema, markedly elevated intracranial pressure or ophthalmic cysticercosis, cysticidal therapy should be avoided as it could worsen the inflammatory reaction. In such circumstances, corticosteroids alone are generally used [1,7]. Adjunctive corticosteroids should be started at least 24 h before cysticidal therapy. Finally, antiparasitic drugs are ineffective on calcified lesions, in this case the treatment is based on symptomatic therapy such as antiseizure medication. Calcified neurocysticercosis could be a cause of chronic epilepsy, which may be refractory in case of hippocampal atrophy [1].

Despite these uncertainties, prompt therapeutic intervention was necessary. The patient presented with severe multifocal CNS lesions, compatible imaging, initial seropositivity, and a life-threatening pyogenic complication. Delaying antiparasitic therapy to pursue further diagnostic confirmation would have carried substantial risk. The subsequent favorable clinical, radiological, and serological evolution supports the appropriateness of a pragmatic, safety-oriented therapeutic strategy under conditions of unavoidable diagnostic ambiguity.

Bacterial superinfection should be suspected when another infectious focus is present, as in our case with concomitant pneumonia, and particularly in patients with potential predisposing factors such as immune dysregulation associated with Down syndrome.

If a bacterial superinfection is suspected, prompt antibiotic treatment is needed. Empiric therapy includes a third-generation cephalosporin, metronidazole and vancomycin [20]. Culture of lesions will guide specific antibiotic treatment.

Occasionally surgery may be required to manage complications like obstructive hydrocephalus or to remove a single lesion.

## 4. Conclusions

This case underscores the diagnostic complexity of suspected neurocysticercosis in non-endemic settings, particularly when the clinical presentation is shaped by host-specific factors, including underlying neurodevelopmental and immunological abnormalities, and by concurrent infectious processes that mask or mimic characteristic features of the disease.

In the absence of histopathological confirmation and with serological limitations, the diagnosis remained presumptive; however, the combination of clinical evolution, neuroimaging features, and microbiological findings provided a coherent working framework. The coexistence of streptococcal superinfection highlights an uncommon but clinically significant complication that can accelerate deterioration and demands early recognition.

Host-related vulnerability, most notably the immune dysregulation associated with Down syndrome, likely contributed both to susceptibility to parasitic infection and to subsequent bacterial invasion, suggesting a potential and underrecognized risk factor for severe or atypical disease trajectories. Epidemiological uncertainty, including the possibility of long-latency infection or unrecognized household exposure, further reinforces the need to maintain neurocysticercosis within the differential diagnosis even in low-prevalence regions.

From a therapeutic standpoint, this case illustrates that timely initiation of combined antimicrobial and antiparasitic therapy may be justified despite diagnostic ambiguity when the risk of progression is high. A safety-oriented, evidence-integrated approach, supported by serial clinical and radiological monitoring, facilitated a favorable outcome in this complex scenario. Continued awareness of atypical presentations, host-specific risk factors, and rare superinfections is essential for optimizing management and improving prognosis in similar cases.

## Figures and Tables

**Table 1:** Clinical timeline of the case.

Time Point	Key Events
**1 month before**	Hospitalization for pneumonia
**Day 0**	Emergency department presentation with drowsiness and altered mental status, followed by cardio-respiratory arrest Brain CT scan: multiple hypodense ring-enhancing lesions, modest mass effect
**Day 1**	ICU admission; initiation of broad-spectrum antimicrobials (ceftriaxone, vancomycin, metronidazole, liposomal amphotericin B) Brain MRI: multiple ring-enhancing lesions with edema and mass effect Neurosurgical intervention (placement of an external ventricular drain and stereotactic drainage); BCID PCR positive for *Streptococcus* spp.
**Day 5**	Positive *Taenia solium* serology. Start albendazole plus praziquantel, after initiation of corticosteroid therapy
**Week 4**	*Taenia solium* serology: borderline
**Week 8**	*Taenia solium* serology: negative
**Week 10**	Progressive clinical and radiological improvement; discharge Total antibiotic therapy duration: 8 weeks Total antiparasitic therapy duration: 4 weeks
**Week 16**	MRI demonstrating progressive radiological improvement

## Data Availability

No new data were created or analyzed in this study.

## Abbreviations

The following abbreviations are used in this manuscript:

CT	Computed tomography
CRP	C-reactive protein
MRI	Magnetic resonance imaging
ICU	Intensive care unit
